# Treatment Interruption after Pregnancy: Effects on Disease Progression and Laboratory Findings

**DOI:** 10.1155/2009/456717

**Published:** 2009-11-02

**Authors:** D. H. Watts, M. Lu, B. Thompson, R. E. Tuomala, W. A. Meyer, H. Mendez, K. Rich, C. Hanson, P. LaRussa, C. Diaz, L. M. Mofenson

**Affiliations:** ^1^Pediatric, Adolescent and Maternal AIDS Branch, Eunice Kennedy Shriver National Institute of Child Health and Human Development, Bethesda, MD 20892, USA; ^2^Clinical Trials and Surveys Corp., Owings Mills, MD 21117, USA; ^3^Brigham and Women's Hospital, Harvard Medical School, Boston, MA 02115, USA; ^4^Quest Diagnostics, Baltimore, MD 21227, USA; ^5^Department of Pediatrics, State University of New York Downstate, Brooklyn, NY 11203, USA; ^6^Department of Pediatrics, University of Illinois, Chicago, IL 60612, USA; ^7^Department of Pediatrics, Baylor University, Houston, TX 77030, USA; ^8^Department of Pediatrics, Columbia University, New York, NY 10032, USA; ^9^Department of Pediatrics, University of Puerto Rico, San Juan, PR 00936, USA

## Abstract

*Objective*. To assess clinical progression and inflammatory markers among women stopping or continuing antiretroviral therapy (ART) after pregnancy. *Methods*. ART-naïve women with CD4+ lymphocyte counts >350 cells/uL initiating ART during pregnancy had clinical events and laboratory markers compared over one year postpartum between those stopping (*n* = 59) or continuing (*n* = 147) ART. *Results*. Slopes in CD4 count and HIV RNA did not differ between groups overall and in subsets of ZDV or combination therapy. The hazard ratio (HR) of a new class B event was 2.09 (95% CI 0.79–5.58) among women stopping ART, 1.24 (0.31–4.95) in those stopping ZDV, and 2.93 (0.64–13.36) among those stopping combination therapy. Women stopping ART had increased immune activation. No significant differences were seen in C-reactive protein, lipids, leptin, or interleukin-6. *Conclusions*. While changes in CD4 and HIV RNA levels over one year were similar between women stopping or continuing ART postpartum, higher immune activation among women stopping therapy requires further study.

## 1. Introduction

Current guidelines recommend that pregnant women be treated with highly active antiretroviral therapy (HAART) regimens for prevention of perinatal transmission of HIV, even if therapy is not yet recommended for treatment of maternal HIV infection [1]. These therapies are generally welltolerated during pregnancy [2] and have resulted in rates of maternal-to-child transmission (MTCT) of under 2% among nonbreastfeeding populations [3]. Antiretroviral therapy is often discontinued after delivery if prepregnancy therapy was not indicated for maternal health [1].

Studies of scheduled treatment interruption showing poorer outcomes among subjects randomized to treatment interruption rather than continuous therapy have raised the question of whether discontinuing therapy after delivery among women receiving HAART for prevention of MTCT may be harmful to the mother. Several small studies using various treatment schedules have not suggested harm from scheduled treatment interruptions, although all have shown lower CD4+ lymphocyte counts at the end of the study in treatment interruption groups [4–6]. Recently however, results from other studies have shown higher rates of morbidity and mortality among subjects assigned to a treatment interruption arm compared to those provided with continuous therapy. The CD4-guided therapy arm of the Trivacan trial in Africa was stopped early because of a significantly increased rate of serious morbidity in the interruption arm (15.2/100 person-years) compared to the continuous therapy arm (6.7/100 person-years, RR 2.27, 95% CI 1.15–4.76) [7]. At enrollment, all subjects had CD4+ cell counts above 350 cells/uL and HIV RNA below 300 copies/mL. Therapy was reinstituted for a CD4 count below 250 cells/uL. The largest trial reported to date, the SMART study, used similar inclusion and therapy interruption/reinstitution guidelines and included 5472 subjects [8]. In SMART, the rate of opportunistic disease or death was 3.3/100 person-years in the therapy interruption group and 1.3/100 person-years in the continuous therapy group (HR 2.6, 95% CI 1.9–3.7 for interruption compared to continuous group). Unexpectedly, the hazard ratio for major cardiovascular, renal, and hepatic disease was 1.7 (95% CI 1.1–2.5) for the interruption compared to the continuous group, despite less overall antiretroviral drug exposure in the interruption group.

While the results of TRIVACAN and SMART raise concerns regarding the potential risk of stopping therapy after use in pregnancy for prevention of perinatal transmission, both of these trials used lower CD4+ lymphocyte count thresholds for resumption of therapy than would be used based on clinical treatment guidelines [9]. Data on outcomes after antiretroviral therapy (ART) limited to pregnancy compared to similar women not receiving therapy or continuing therapy are limited and primarily reflect use of zidovudine (ZDV) monotherapy rather than HAART [10–15]. Given the paucity of data on the long-term maternal effects of short-term HAART regimens for prevention of perinatal transmission of HIV and the concerns regarding therapy interruption raised by the SMART and TrivAcan studies, we compared clinical and laboratory outcomes after delivery among women enrolled to the Women and Infants Transmission Study (WITS), a longitudinal cohort study, who either stopped or continued antiretroviral therapy at delivery that had been started for prevention of MTCT of HIV. Laboratory markers of immune activation and potential cardiovascular and metabolic risk were included.

## 2. Methods

WITS is a multicenter prospective observational study of pregnant women and their infants [16]. Beginning in December (1989), pregnant HIV-1-infected women were recruited at centers in Chicago, Massachusetts (Boston and Worcester), New York City, and San Juan. Sites were added in Brooklyn in 1991 and in Houston in 1993. The study was approved by each site's Institutional Review Board, and all women provided informed consent for enrollment of themselves and their infants. Women were enrolled during pregnancy or within 7 days after delivery, and infants were enrolled within 7 days of birth. For women monitored during pregnancy, study visits occurred at or before 20 weeks' gestation, at 25 ± 2 weeks, at 32 ± 2 weeks, and at delivery. Women were followed for one year after delivery with visits at two, six, and twelve months. At each visit, a detailed medical and behavioral questionnaire was administered, and a physical examination and phlebotomy were performed. Additional data on women and infants were obtained from medical record abstraction.

Antiretroviral treatment was not prescribed as part of the study but detailed data on its use was collected. Therapy during pregnancy was categorized as ZDV monotherapy or combination therapy, consisting of at least two nucleoside reverse transcriptase inhibitors with or without nonnucleoside reverse transcriptase inhibitors or protease inhibitors. Clinical events were categorized using the 1993 CDC revised surveillance case definition [17].

To be eligible for inclusion in this analysis, women had to be enrolled after 6/1/1994 when use of ZDV for prevention of HIV transmission became standard therapy for pregnant women and before 6/30/2006 to allow a year of followup postpartum. In addition, they had to be enrolled by 32 weeks of gestation to allow time for antiretroviral therapy to be administrated and be ART naïve at enrollment. They had to have a CD4+ lymphocyte count above 350 cells/uL at enrollment so that ART was being prescribed primarily for prevention of MTCT and not for maternal health indications. Of the 3297 women enrolled to WITS by 6/30/2006, 2777 had a confirmed delivery, 1165 were ART naïve at enrollment, and 1091 of these were enrolled before the delivery visit. Of these, 512 received ART during pregnancy, 261 of whom had a CD4+ lymphocyte count above 350 cells/uL at enrollment during pregnancy, and 206 were enrolled after 6/1/1994. The 206 women included did not differ significantly from the 3091 women not included in age, race/ethnicity, history of CDC class B or C condition, or HIV RNA levels. Based on inclusion criteria, included women had higher CD4+ lymphocyte counts (mean 603 cells/uL versus 467 cells/uL, *P* < .001) and lower gestational age at enrollment (16.1 weeks versus 21.6 weeks, *P* < .001) than the excluded women.

To assess HIV disease progression, we used knotted splines and mixed effect models to compare the post delivery slope of the CD4+ lymphocyte counts, CD4+ lymphocyte percents, and HIV RNA levels between women continuing or stopping ART at delivery using [18, 19]. Rates of development of new CDC class B or C events were compared using Cox proportional hazard models [20]. The marginal mean values for laboratory testing postpartum were compared between the above listed groups using a general estimating equation [19]. When indicated, some data were log-transformed to produce a normal distribution.

CD4+ lymphocyte counts were determined by flow cytometry at DAIDS Immunology Quality Assurance Program certified laboratories. Lymphocyte phenotyping was done using a FACScan (Becton Dickinson, San Jose, CA) equipped with LYSIS II software as previously described [21]. Plasma HIV-1 RNA was measured in stored specimens using the Roche Amplicor HIV-1 Monitor Test (Roche Diagnostic Systems, Branchburg, NJ), as described in [22]. Specimens were assayed for routine metabolic analytes (total cholesterol, high-density lipoprotein (HDL) cholesterol, triglycerides, high sensitivity C-reactive protein (hsCRP)) at Quest Diagnostics, Baltimore, MD and for specialty analytes (leptin, lipoprotein-associated phospholipase A2 (Lp-PLA2), interleukin 6 (IL-6)) at Quest Diagnostics Nichols Institute, San Juan Capistrano, CA. Aliquots of frozen serum were shipped to Quest Diagnostics on dry ice for batch testing of each analyte. Low-density lipoprotein cholesterol (LDL) was calculated using the Friedewald Equation for those samples exhibiting triglyceride values below 400 mg/dL. The laboratory utilized the FDA-cleared Cholesterol Esterase/Oxidase method of Olympus (Olympus America, Inc., Melville, NY) on an automated testing platform for total cholesterol quantitation. For HDL cholesterol, the laboratory utilized the FDA-cleared, HDL-Cholesterol Plus 2nd Generation assay from Roche Diagnostics (Indianapolis, IN) on an automated testing platform. For triglyceride quantitation, the laboratory utilized the FDA-cleared Triglyceride reagents from Olympus on an automated testing platform. Serum high sensitivity CRP levels were measured using the FDA-cleared, automated BN II in vitro diagnostic system (Dade Behring, Inc., Newark, DE). This method uses a particle enhanced immunonephelometric assay with a sensitivity of 0.2 mg/L. Leptin was measured using the Research Use Only Human Leptin RIA Kit from Linco Research, Inc. (St. Charles Missouri). This method is a competitive radioimmunoassay that utilizes a fixed concentration of Iodine-125-labeled human leptin tracer antigen with a constant dilution of human leptin antiserum. The concentration of unknown human leptin in the study subject's samples is determined against a calibration curve that is set up with each assay run that includes increasing concentrations of standard unlabeled human leptin antigen. Human IL-6 was measured with the Research Use Only Quantikine HS Human IL-6 Immunoassay kit from R&D Systems, Inc. (Minneapolis, MN). This assay utilizes a quantitative sandwich enzyme immunoassay technique to estimate the concentration of IL-6 in serum specimens. Lp-PLA2 was measured with the FDA-approved PLAC test kit from diaDexus (South San Francisco, CA).

## 3. Results

Of the 206 women eligible for inclusion, 147 continued therapy after delivery and 59 discontinued. A comparison of the two groups is shown in Table 1. Women stopping therapy at delivery were slightly younger and had higher CD4+ lymphocyte counts than women continuing therapy, but the groups were similar in race/ethnicity, gestational age at enrollment, history of class B or C illness, and HIV RNA levels.

ZDV monotherapy was used by 103 women during pregnancy, 41 of whom stopped therapy at delivery, and 62 of whom continued. Three additional women received monotherapy with other drugs, one with nevirapine and two with didanosine. One hundred women took two or more antiretroviral drugs during pregnancy, 18 of whom stopped therapy at delivery, and 82 of whom continued. Of the women on combination therapy, 27 took two NRTI's, two took triple NRTI regimens including abacavir, 11 took two NRTI's plus an NNRTI, 55 took two NRTI's with one or more PI's, four took triple class regimens, and one took a regimen of one NRTI with an NNRTI.

**Figure 1:**
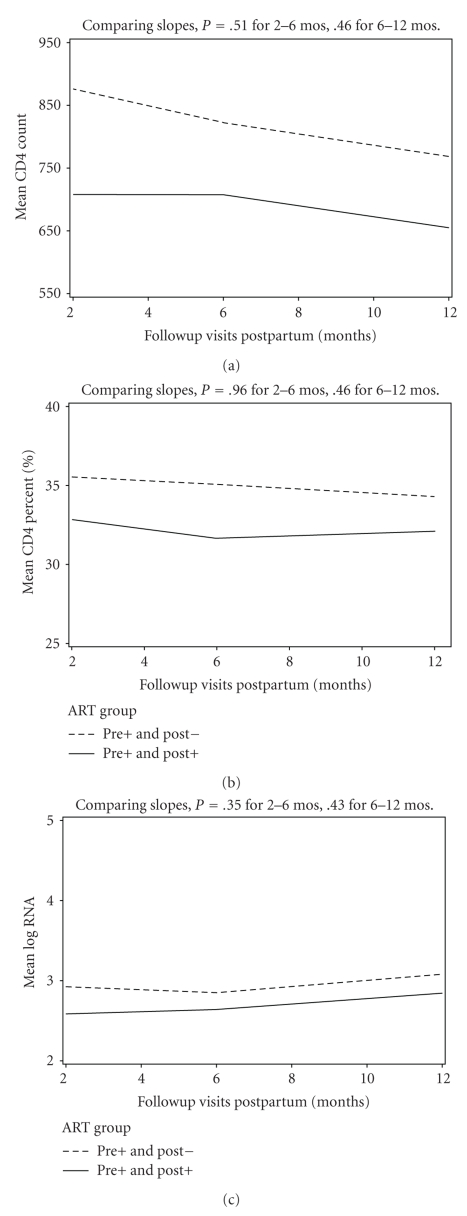
Changes in (a) CD4+ lymphocyte count, (b) CD4+ lymphocyte percentage, and (c) HIV RNA levels between two months and 12 months after delivery among all women (regardless of antiretroviral regimen) stopping (dashed line) or continuing (solid line) antiretroviral therapy after delivery.

We compared the risk of HIV disease progression between women who stopped and women who continued therapy after delivery in several ways. The slopes of CD4+ lymphocyte counts and percentages and of HIV RNA levels were compared between two and six months and six and twelve months postpartum to assess for “rebound changes” after stopping therapy. As shown in Figure 1 and Table 2, the rate of change in CD4+ cell measures and HIV RNA was not significantly different between the two groups. To evaluate further by type of therapy during pregnancy, the changes in CD4+ lymphocyte count and percentage and HIV RNA level were compared among those stopping or continuing ZDV monotherapy and those stopping or continuing combination regimens. Table 2 shows the difference in slope for each parameter between all women who stopped or continued therapy, women who stopped or continued ZDV monotherapy, and those who continued or stopped combination therapy. For example, over two to six months after delivery, women in the entire group who stopped therapy had a 12.2 cells/month loss in CD4+ lymphocyte count compared to women continuing therapy, but the difference was not significant. No significant differences between slopes were found for any of the time periods or any of the therapy groups.

Clinical progression was also assessed. None of the women developed a new class C condition during the first year postpartum. Among the entire group, the hazard ratio (HR) for development of a new class B condition was 2.09 (95% CI 0.79–5.58, *P* = .14) for women who stopped therapy at delivery compared to those who continued. For the ZDV monotherapy group, the HR was 1.24 (95% CI 0.31–4.95, *P* = .76) and for the combination therapy group the HR was 2.93 (95% CI 0.64–13.36, *P* = .16). Class B events among women stopping therapy included cervical dysplasia/carcinoma in situ (CIS) in four, recurrent pneumonia in one, and vaginal yeast infection for over 28 days in one. Class B events among women continuing therapy included cervical dysplasia/CIS in three, oropharyngeal candidiasis in two, other infections in two, and other gastrointestinal problem, vaginal yeast infection for over 28 days, and pelvic inflammatory disease in one each.

Since treatment interruption studies have suggested an increased risk of morbidity not specifically related to HIV disease progression, we evaluated several laboratory markers of immune activation and of potentially increased risk of cardiovascular disease (Table 3). CD4+ and CD8+ counts and percentages are shown for all women in the analysis and each subgroup. Lymphocyte activation markers were available for a subset of women. Of note, the CD8+CD38+ T lymphocyte and CD8+DR+ T lymphocyte percentages were higher in women stopping therapy than in those continuing in the overall group and the ZDV group. Results were available in too few women in the combination therapy group to allow meaningful analyses. HS-CRP, lipid levels, leptin, and interleukin-6 levels were not different between the groups among all women and those receiving ZDV. Lp-PLA2 levels were significantly different in the total group and the combination therapy group, but levels were higher in the women continuing therapy compared to those stopping.

## 4. Discussion

Overall, the data on changes in CD4+ lymphocyte count, HIV RNA levels, and clinical progression are reassuring over the first year postpartum among women with CD4+ lymphocyte counts above 350 cells/uL who choose to stop antiretroviral therapy after delivery compared to those who continue. One recent retrospective review found an increased risk of death or opportunistic infection among women stopping therapy after delivery, but the group was more heterogeneous than in the current study with 46% having previous ARV exposure and 36% having a pre-ARV CD4+ cell count below 350 cells/uL [15]. The number of Class B events in our study was low for both the continuation and noncontinuation groups, making it difficult to further define the risk of developing these events according to treatment continuation. The relative risk estimate for combination therapy is consistent with other studies and is consistent with changes in activated CD8+ counts and percents that was seen in this study. However, when taken in the context of the overall low numbers of events, and insignificant changes in CD4+ count and CD8+ count slopes there is little evidence that stopping therapy increases a woman's risk of progression. Among those enrolled to the SMART study, divergence of the curves in the risk of opportunistic infection or death were seen by 12 months after enrollment between the drug conservation and viral suppression groups, even among those who were therapy naïve or not on antiretroviral therapy at delivery [8, 23]. However, women included in the current study were all therapy naïve with nadir CD4+ lymphocyte counts above 350 cells/uL and baseline CD4+ cell counts of 603 cells/uL, compared to the SMART study in which the median nadir CD4+ lymphocyte count was 250 cells/uL and participants had initiated antiretroviral therapy a median of six years before enrollment [8]. Among subjects in SMART who were antiretroviral naïve, the nadir CD4+ lymphocyte count was 376 cells/uL and the baseline was 437 cells/uL, also lower than in the current study [23]. Thus, differences in disease progression in those stopping therapy compared to those continuing may take longer to manifest among women with higher baseline CD4+ cell counts and no prior antiretroviral therapy.

A concerning laboratory finding between the groups was higher levels of CD8+ cell activation among women stopping therapy at delivery compared to those continuing. Pregnancy has been shown to lead to increased levels of CD8+DR+CD38+ lymphocytes in HIV-uninfected women compared to nonpregnant women, but levels return to baseline after delivery [21, 24], while levels are much higher during pregnancy in HIV-infected women and remain elevated postpartum in the absence of antiretroviral therapy [21]. The current data suggest that continuing antiretroviral therapy after pregnancy may reduce levels of CD8+ cell activation among HIV-infected women overall and among women on ZDV compared to women who stop therapy, but lymphocyte activation results were available for only two women stopping combination regimens, precluding a comparison in this group. Increased levels of CD8+DR+CD38+ cells have been associated with both an increased risk of disease progression [25] and reduced CD4+ cell gains on HAART [26]. Thus persistently high levels postpartum may predict more rapid disease progression over time in those stopping therapy, but longer followup is required to assess this hypothesis. In addition, lymphocyte activation should be compared between women stopping and continuing HAART regimens after delivery in larger numbers of women.

Elevated hsCRP has recently been shown to predict HIV disease progression among untreated HIV-infected women postpartum [27]. We did not observe significant differences in CRP levels between women stopping versus continuing therapy postpartum, suggesting against differential risk in disease progression.

One of the areas of increased morbidity and mortality noted in subjects in the drug conservation group of the SMART trial was cardiovascular outcomes including myocardial infarction, coronary artery disease requiring surgery, and stroke [8]. Since the median age in the SMART trial was 43 years compared to 27 years in the current study, we evaluated surrogate markers of cardiovascular risk since cardiovascular events were not expected. Median values of laboratory markers that have been associated with an increased risk of cardiovascular outcomes, including elevated cholesterol, low density lipoproteins, triglycerides, and high sensitivity C-reactive protein, and decreased high density lipoproteins were not different between groups continuing versus stopping therapy. These results do not suggest an increased risk of cardiovascular morbidity among women stopping therapy but the predictive value of these markers in HIV-infected populations has primarily been assessed in populations of older men [28], and gender differences have been noted in some analytes in the general population [29]. The only analyte found to differ between the groups was LP-PLA2, a macrophage-derived enzyme that has been an independent predictor of cardiovascular events in healthy populations [30, 31]. Lp-PLA2 is not an acute phase reactant and is only minimally correlated with systemic inflammatory and hemostatic markers, suggesting that it may be useful for prediction of cardiovascular risk in HIV-infected groups, although further study is required [31]. Of note, Lp-PLA2 levels were higher among women continuing therapy, possibly indicating higher risk associated with continued ART exposure or possibly related to the older age. The median values in both groups were lower than the proposed cutoff of below 235 ng/mL, so the clinical implications of the differences require further study. The overall results however do not suggest a major difference in cardiovascular risk between the groups, and especially not increased risk among women stopping therapy.

While the SMART study clearly showed benefits to continuing ART once initiated, not all STI studies have shown differences in outcome. Negative studies had smaller sample sizes but also used higher thresholds for restarting ART. Studies using a threshold of 350 cells/uL for reinitiating ART tended to show fewer differences in outcomes between continuous versus interrupted therapy [4–6]. These studies are more consistent with the current standard of care in developing countries, with initiation of therapy recommended at a CD4+ cell count of 350 cells/uL, which would apply to pregnant women stopping ART used for prevention of PMTCT. In developing countries, therapy is usually initiated at a lower CD4+ cell count of 200–250 cells/uL, more similar to the situation in the SMART study. In addition, if studies currently in progress confirm reduced transmission with maternal HAART during breastfeeding, women in developing countries may have longer periods of HAART for prevention of PMTCT before discontinuation, potentially increasing the risk of adverse outcomes with discontinuation. Assessment of the effects of discontinuing ART used for PMTCT must be included in studies in resource limited settings.

The current results are generally reassuring that stopping ART used solely for PMTCT does not increase the short-term risk of HIV disease progression or of abnormalities in common cardiovascular risk markers. However, followup was only for one year postpartum, and longer followup may be needed to detect differences in this relatively healthy population [15]. Women were not randomized, but chose whether or not to continue therapy in consultation with their health care provider. Thus, there may be unmeasured confounders that affected the outcomes and mitigated differences. Finally, the numbers included in this study were relatively low, especially in the group stopping combination therapy, thus limiting power.

## 5. Conclusions

Overall, the data do not indicate major differences in short-term outcome between women with CD4+ cell counts above 350 cells/uL who choose to stop or continue ART that was initiated for PMTCT. However, declines in lymphocyte activation markers among women continuing ART are consistent with large treatment interruption studies demonstrating improved outcomes with continuous rather than intermittent ART. As use of combined ART regimens for prevention of PMTCT expands, especially in developing countries where thresholds for reinitiation of therapy are lower and duration of preventive therapy may be longer if used during breastfeeding, further evaluation, in randomized trials, of the risks and benefits of continuing therapy, is required.

## Figures and Tables

**Table 1:** Baseline characteristics of women included in the study according to status of antiretroviral therapy after delivery.

Characteristic	Total *n* = 206	Stopped therapy *n* = 59	Continued therapy *n* = 147	P*
Race/ethnicity				.43
* *White	22 (10.7%)	8 (13.6%)	14 (9.5%)	
* *African American	98 (47.6%)	29 (49.2%)	69 (46.9%)	
* *Latina	75 (36.4%)	21 (35.6%)	54 (36.7%)	
* *Other	11 (5.3%)	1 (1.7%)	10 (6.8%)	
CD4+ lymphocyte count				.03
* *350–500 cells/uL	84 (40.9%)	17 (28.8%)	67 (45.6%)	
* *>500 cells/uL	122 (59.2%)	42 (71.2%)	80 (54.4%)	
CDC Classified events				
* *Class B or worse	59 (28.6%)	15 (25.4%)	44 (29.9%)	.52**
* *Class C	6 (2.9%)	2 (3.4%)	4 (2.7%)	.80**
Mean enrollment CD4+ lymphocyte count	603	680.3 ± 63.9	572.5 ± 30.5	.004
Mean enrollment CD4+ percentage		34.2% ± 1.9	31.8% ± 1.4	.06
Mean gestational age (weeks)	16.1	16.1 ± 1.9	16.1 ± 1.2	.97
Mean enrollment HIV RNA (log10)		2.63 ± 0.5	3.00 ± 0.3	.23
Mean maternal age		25.9 ± 1.2	27.7 ± 1.0	.04
Therapy during pregnancy				
* *Zidovudine monotherapy^⋀^	103 (50%)	41 (40%)	62 (60%)	
* *Combination therapy	100 (49%)	18 (18%)	82 (82%)	

* T test comparing means or proportions except as noted. ** Chi square test used. ^⋀^ Three additional women received monotherapy with other agents, one with nevirapine and two with didanosine.

**Table 2:** Difference in slope and *P*-values between women stopping or continuing therapy after delivery, according to antiretroviral therapy use during pregnancy.

	All women		ZDV monotherapy		Combination therapy	
*Parameter*	*2–6 months*	*6–12 months*	*2–6 months*	*Parameter*	*2–6 months*	*6–12 months*
CD4 count	−12.2, *P* = .51	10.0, *P* = .46	−19.5, *P* = .52	CD4 count	−12.2, *P* = .51	10.0, *P* = .46
CD4+ %	−0.01, *P* = .96	−0.13, *P* = .46	0.06, *P* = .82	CD4+ %	−0.01, *P* = .96	−0.13, *P* = .46
Log10 HIV RNA	−0.07, *P* = .35	−0.04, *P* = .43	−0.10, *P* = .25	Log10 HIV RNA	−0.07, *P* = .35	−0.04, *P* = .43

**Table 3:** Median postpartum laboratory values among women continuing or stopping antiretroviral therapy postpartum according to therapy during pregnancy.

	All women			ZDV monotherapy			Combination therapy		
*Parameter*	+/-	+/+	*P*	+/-	+/+	*p*	+/-	+/+	*P*
CD4+ lymphocyte count	733.2	701.7	.47	725.2	711.0	.84	754	690.5	.33
CD4+ lymphocyte %	34.8	33.9	.42	34.2	32.6	.31	36.3	34.9	.44
CD8+ lymphocyte count	935.1	949.9	.81	985.3	1042.8	.56	818.4	874.0	.48
CD8+ lymphocyte %	44.7	44.7	.98	46.4	48.1	.41	40.8	41.9	.67
CD19+ %	9.0	9.6	.72	9.6	8.9	.72			
CD8+CD57+ %	17.3	16.6	.80	18.8	18.8	.99			
CD8+DR+ %	**36.3**	**26.6**	**.02**	**35.9**	**24.9**	**.01**			
CD16+CD56+ %	**4.8**	**7.4**	**.04**	5.2	7.8	.12			
CD8+CD38+ %	**47.6**	**36.7**	**.001**	**49.8**	**40.5**	**.04**			
High sensitivity C-reactive protein (mg/L)	2.8	3.0	.69	2.7	3.1	.58			
Cholesterol (mg/dL)	177.7	188.7	.10	177.7	188.7	.11			
High density lipoprotein (mg/dL)	47.8	49.3	.56	48.1	49.2	.67			
Low density lipoprotein (mg/dL)	95.6	101.5	.30	94.6	100.5	.35			
Triglycerides (mg/dL)	180.0	184.7	.81	181.0	187.1	.77			
Leptin (ng/dL)	21.0	19.3	.54	20.1	19.7	.90			
Interleukin-6 (pg/dL)	1.9	1.6	.22	2.2	1.7	.33	1.7	1.6	.65
Lipoprotein phospholipase A-2 (ng/dL)	**166.1**	**187.0**	**.05**	170.6	188.5	.37	**161.3**	**185.5**	**.05**

+/- indicates women who stopped therapy at delivery; +/+ indicates women who continued therapy after delivery. Postpartum specimens were collected at six to 12 months postpartum.
